# Male-biased sex ratio does not promote increased sperm competitiveness in the seed beetle, *Callosobruchus maculatus*

**DOI:** 10.1038/srep28153

**Published:** 2016-06-16

**Authors:** Kathryn B. McNamara, Stephen P. Robinson, Márta E. Rosa, Nadia S. Sloan, Emile van Lieshout, Leigh W. Simmons

**Affiliations:** 1 Centre for Evolutionary Biology, School of Animal Biology (M092), the University of Western Australia, Crawley, 6009, Australia; 2 Department of Evolutionary Zoology and Human Biology, University of Debrecen, Debrecen, H-4032, Hungary; 3 Department of Ecology, Szent István University, Budapest, H-1077, Hungary

## Abstract

Sperm competition risk and intensity can select for adaptations that increase male fertilisation success. Evolutionary responses are examined typically by generating increased strength of sexual selection via direct manipulation of female mating rates (by enforcing monandry or polyandry) or by alteration of adult sex ratios. Despite being a model species for sexual selection research, the effect of sexual selection intensity via adult sex-ratio manipulation on male investment strategies has not been investigated in the seed beetle, *Callosobruchus maculatus.* We imposed 32 generations of experimental evolution on 10 populations of beetles by manipulating adult sex ratio. Contrary to predictions, males evolving in male-biased populations did not increase their testes and accessory gland size. This absence of divergence in ejaculate investment was also reflected in the fact that males from male-biased populations were not more successful in either preventing females from remating, or in competing directly for fertilisations. These populations already demonstrate divergence in mating behaviour and immunity, suggesting sufficient generations have passed to allow divergence in physiological and behavioural traits. We propose several explanations for the absence of divergence in sperm competitiveness among our populations and the pitfalls of using sex ratio manipulation to assess evolutionary responses to sexual selection intensity.

Female multiple mating is ubiquitous across the animal kingdom. A corollary of this phenomenon is that males must compete with other males for access to both females and fertilizations^1^,^2^. In response to this postcopulatory sexual selection, a suite of morphological, physiological and behavioural traits have been identified in males that increase paternity share (reviewed in^3^). Males, for example, can increase their sperm competitive success by increasing their testes^4^ and/or accessory gland size^5^, increasing their sperm quality^6^,^7^ and/or quantity, or by preventing or delaying remating by females^8^.

One widespread response to postcopulatory sexual selection is for males to modulate ejaculate production. Doing so, however, involves non-trivial costs for males^9^,^10^. In response to these costs, males strategically provision ejaculates according to both sperm competition risk (the likelihood that female mating partners have or will mate multiply) and sperm competition intensity (the number of males with which a female mates). Theory predicts that when the strength of selection from sperm competition is high and mating opportunities are rare males should increase their investment into testes size, sperm quality or number, and thus, sperm competitiveness^3^,^11^. A range of empirical studies and meta-analyses of these studies have provided evidence that males of many taxa are able to respond plastically to socio-sexual cues to adjust strategically their ejaculate investment^3^,^10^,^12^,^13^. Moreover, comparative studies of multiple taxa clearly demonstrate evolutionary increases in male reproductive investment (testes size) across generations with increasing selection from sperm competition risk and intensity^14^,^15^,^16^,^17^.

Experimental evolution is an important tool to examine male evolutionary responses to changes in the strength of postcopulatory sexual selection within species^18^. This technique, in which female mating frequency is manipulated either directly by enforcing monandry/polyandry or indirectly by altering adult sex ratios, has been used in a variety of taxa to demonstrate evolutionary changes in ejaculate expenditure in response to postcopulatory selection^19^,^20^,^21^,^22^,^23^,^24^. For example, in *Drosophila melanogaster* there is evidence of increased male reproductive investment in response to increased postcopulatory selection from changes in both mating frequency^21^ and sex ratio manipulation^22^.

Although sex ratio manipulation has been used extensively as a means to change sexual selection intensity, this approach has not been investigated in the cowpea seed beetle, *Callosobruchus maculatus*. This beetle is an important model species for the study of sexual selection and is ideal for examining evolved responses to postcopulatory sexual selection^25^,^26^,^27^. Previous studies have demonstrated that variation in male ejaculate investment affects female behaviour and reproductive output. For example, females mated to virgin males remate less readily and are more fecund than when mated to non-virgin males, although they also show reduced longevity^28^. Importantly, laboratory populations evolving under polyandry for 90 generations had males with larger testes than populations evolving under enforced monandry^27^. Thus, male *C. maculatus* increase reproductive investment in response to mating frequency manipulation. However, whether increased selection intensity via manipulation of adult sex ratio also affects male reproductive investment is unknown.

In the present study, we experimentally manipulated sex ratio in laboratory populations of *C. maculatus* to examine potential changes in reproductive investment of males. Specifically, males evolved under either female-biased or male-biased population sex ratio (low or high sexual selection intensity respectively). After 32 generations of experimental evolution, we tested for male sperm competitiveness or their ability to induce a refractory period in females. Previously, we reported evolutionary divergence in immune function and mating behaviour among these populations^26^. We predicted that the increased selection intensity in male-biased populations would generate increased sperm production (testes size) or seminal fluid investment, and that this would result in increased sperm competitiveness by either directly increasing a male’s paternity share or increasing the female’s non-receptive period.

## Materials and Methods

### Experimental evolution lines

Experimental evolution lines were founded using beetles sourced from a large outbred population (hereafter referred to as the stock population)^26^ that originated from a stock culture held by CSIRO (Canberra, Australia). Experimental evolution treatments manipulated the sex ratio of the population (80:40 or 40:80 males:females). We haphazardly assigned virgin individuals to 5 replicate female-biased or five replicate male-biased populations. Female-biased populations received 200 g of mung beans (*Vigna radiata*) whereas male-biased populations received 100 g, to avoid differences in larval competition between treatments. Populations were maintained at 30˚C under 12 h:12 h light:dark. Offspring were obtained by isolating 300 beans into 1.5 mL microtubes 24 h following the first observed adult emergences in each line. Once sufficient virgin adults had emerged, typically after two days, we established new sex-biased populations (as above). After 32 generations, we subjected each line to one generation of relaxed selection (equal sex ratios) before assessing changes in sexually selected traits. We did this to reduce any non-genetic parental effects that might potentially influence the traits measured.

We maintained stock males and females under the same light and temperature conditions as the experimental lines. To create populations of stock individuals we removed adults from our large source stock population (comprised of thousands of adults and developing larvae on ad libitum beans) and placed approximately 300 adults (likely non-virgin) on approximately 200 g of mung beans. This was repeated for approximately 6 containers.

We obtained individuals for mating trials by isolating beans from the selected lines and stock populations into pinhole-ventilated 1.5 mL microtubes. These were checked twice daily and newly emerged, virgin beetles were place individually into a different 1.5 mL microtube. All focal individuals were weighed prior to experimentation, and their post-emergence age recorded. All matings took place in 1.5 mL microtubes, unless otherwise stated.

### Does postcopulatory sexual selection affect a male’s ability to induce a female refractory period?

To examine the ability of males of different sex ratio backgrounds to increase the duration of the female’s refractory period, we mated virgin males (1 day old) from both male-biased and female-biased lines to a single, virgin stock female (1 day old). To measure the weight of the ejaculate transferred to the female, we weighed females to 0.01 mg immediately before and after copulation on a Sartorius SE3 microbalance. We isolated mated females in 1.5 mL microtubes with 4 mung beans to encourage oviposition and remating. The following day, females were provided with a virgin, stock male (1 day old) and given the opportunity to mate. Couples were given 10 mins to mate. If a mating did not occur in this time period, the female was isolated, and the male discarded. Females that did not remate were given the opportunity to remate with another virgin, stock male (1 day old) on the next day. This was repeated until the female remated or for a total of 4 mating attempts. The day on which a female remated, or if she failed to remate was recorded. All mating trials were conducted during the light phase at approximately 25 °C.

### Does postcopulatory sexual selection affect investment into testes and accessory glands?

To examine the effect of sex ratio background on male investment into reproductive morphology, we froze virgin males from each replicate at −20 °C on the day of emergence. We dissected and removed the male’s testes and accessory glands (see^29^ for a drawing of male reproductive morphology). The two largest mesadenia glands were used in measurements as they were the only glands able to withstand measurement without breaking. To standardize the thickness of the male reproductive tract, we laid the testes and accessory glands flat on a haemocytometer. A coverslip was firmly applied so that there was no gap between the coverslip and the cover glass support of the haemocytometer, thus ensuring that the testes and accessory glands were compressed to the same thickness. We took a digital image (x200 magnification) and measured the area of the testes and the accessory glands using the outline tool in ImageJ (version 1.48).

### Does postcopulatory sexual selection affect male sperm competitiveness?

To examine the effect of male sex ratio background on offensive sperm competitiveness, we mated males from each of the sex-ratio lines to a female who had mated once previously. Paternity was assigned using the sterile male technique^30^. Here, a virgin stock female was first mated to a 1 day old, virgin, stock male. Stock males had been sterilized with a 60 Gy dose of radiation from a Cobalt-60 source, under nitrogen anesthesia (5 L min^−1^), 24 h prior to mating (for detailed methods, see^31^). We isolated mated females in 1.5 mL microtubes with 4 mung beans to encourage oviposition and remating. Forty-eight hours later, we provided the female with a male from one of the sex-ratio treatment populations. We placed twice-mated females in 55 mL plastic vials with approximately 30 mung beans and allowed them to oviposit until their deaths. For each female, we counted the number of eggs on the surface of the bean (fecundity) and the number of adult offspring that emerged from the beans approximately 28 days later (fertility).

As irradiated sperm remain functionally competent but contain DNA mutations resulting in early embryonic death, unhatched eggs were attributed to the irradiated male, and hatched offspring to the non-irradiated male. For both copulations, we recorded the duration of the copulation. Copulation duration was defined as commencing when the male had inserted his aedeagus and reclined so that his body and forelegs no longer rested on the female’s back. Copulation ceased when the male removed his aedeagus. We excluded females that did not remate from subsequent analysis.

### Statistics

We conducted all analyses in R 3.0.1. We used mixed-effects models (package ‘lme4’) to account for the identities of replicate evolution lines. We analysed the duration of the female refractory period with an ordinal mixed model (package ‘ordinal’). An ordinal model accounted for the fact that remating success was recorded for only a subset of the female’s lifespan, and was thus not truly continuous. We analysed fecundity, testes/accessory gland size and mating duration data with general linear mixed models. We analysed P_2_ data with a generalised linear mixed model, using a binomial distribution (with a logit link function), with the total number of eggs laid as the binomial denominator. An observation-level random factor was included in the model, to address overdispersion.

We corrected mean and standard error P_2_ values (presented in Table 1) (following^30^) to account for the residual infertility and fertility of irradiated and normal males, respectively. We used published residual fertility (0.06%) and infertility (0.76%) values derived from single matings with irradiated and normal males from the same stock populations as used here (see^31^). Although the use of single matings to acquire residual fertility data could potentially overestimate natural residual infertility, this does not affect our main sperm competition analysis, in which the focal male was always non-irradiated and always mated in the final position. Moreover, our focus was on the relative competitiveness of our treatment lines and not the absolute values of P_2_.

For all models, we optimally power transformed all dependent variables to maximize normality of residuals, and the exponents used were noted with every analysis. We removed non-significant interactions from final models^32^. We included male body weight in all models as it was not affected by sex ratio treatment (mean weight (mg) ± standard error): male-biased = 3.44 ± 0.07, female-biased = 3.29 ± 0.07; Χ^2^_1_ = 1.55, n = 129, P = 0.21).

## Results

### Effects on refractory period

The population sex ratio from which a male was derived did not affect his ability to prevent a female from remating (proportion remating (mean ± standard error): male-biased = 0.82 ± 0.03, female-biased = 0.86 ± 0.05; Χ^2^_1_ = 0.06, P = 0.80). The likelihood that a female remated was also not affected by the male’s weight (Χ^2^_1_ = 0.004, β = 0.02 (0.41), P = 0.95), the female’s weight (Χ^2^_1_ = 1.40, β = 0.29 (0.24), P = 0.24) or the weight of the ejaculate received by the female (Χ^2^_1_ = 0.0001, β = 0.03 (3.54), P = 0.99).

Similarly, the duration of the female refractory period was not influenced by the population sex ratio from which her mate was derived (z = 0.006, P = 0.99; Table 1), male weight (z = 1.22, β = 0.38 (0.31), P = 0.22), female weight (z = −1.24, β = −0.22 (0.18), P = 0.22) or the weight of the ejaculate received by the female (z = 0.90, β = 2.65 (2.95), P = 0.37).

Ejaculate weight was not affected by the population sex ratio from which a male was derived (Χ^2^_1_ = 0.12, P = 0.72; Table 1). Ejaculate weight, however, increased with male weight (Χ^2^_1_ = 8.07, β = 0.03 (0.009), P = 0.004) and copulation duration (Χ^2^_1_ = 34.76, β = 0.00005 (0.00002), P < 0.001), but decreased when males mated with heavier females (Χ^2^_1_ = 8.49, β = −0.016 (0.005), P = 0.003).

### Effects on testes and accessory glands

*Callosobruchus maculatus* males have two bi-lobed testes, but during dissection some were damaged; four lobes were measured in 135 males, three in 55 males and two in 10 males. Mean testes lobe size was used in analysis. In contrast, the total area of the two largest mesadenia glands were used for analysis of accessory gland investment. Testes size was not affected by the population sex ratio from which a male was derived (Χ^2^_1_ = 0.29, P = 0.59; Table 1), but was larger in heavier males (Χ^2^_1_ = 45.70, β = 0.08(0.01), P < 0.001). Similarly, accessory gland size was also not affected by population sex ratio (Χ^2^_1_ = 1.45, P = 0.23; Table 1), but was larger in heavier males (Χ^2^_1_ = 34.76, β = 0.11 (0.02), P < 0.001).

### Does population sex ratio affect male sperm competitiveness?

Two females did not lay eggs and were excluded from subsequent analyses. Five females laid only infertile eggs (females mated to male-biased males = 3; females mated to female-biased males = 2); these data were also excluded from analysis (see^33^). The proportion of offspring sired by the second focal male (P_2_) was not affected by the population sex ratio from which the focal male was derived (Χ^2^_1_ = 0.80, P = 0.37; Table 1), the weight of the focal male (Χ^2^_1_ = 1.52, β = 0.02 (0.02), P = 0.22), the weight of his competitor (Χ^2^_1_ = 0.05, β = −0.01 (0.03), P = 0.82), the mating duration of the focal male (Χ^2^_1_ = 0.32, β = −0.02 (0.04), P = 0.57), or the mating duration of his competitor (Χ^2^_1_ = 0.08, β = −0.01 (0.04), P = 0.77). A non-significant interaction between the mating duration of the focal and competitor male was removed from the final model (Χ^2^_1_ = 0.14, P = 0.70).

For both male-biased and female-biased treatments, the mean corrected P_2_ differed from 0.5, indicating last-male sperm precedence (male-biased lines: t_1,59_ = 6.64, P < 0.0001; female biased lines: t_1,61_ = 4.29, P < 0.0001; Table 1).

The number of eggs laid by a female (including those eggs laid after her first mating) (raised by exponent 0.12) increased with the weight of the focal male (Χ^2^_1_ = 4.88, β = 0.59 (0.27), P = 0.03), and the weight of the female (Χ^2^_1_ = 21.33, β = 1.22 (0.26), P < 0.01). Fecundity, however, was not affected by the population sex ratio from which the focal male was derived (Χ^2^_1_ = 0.70, P = 0.40; Table 1), the weight of his competitor (Χ^2^_1_ = 0.39, β = −0.24 (0.39), P = 0.53), the mating duration of the focal male (Χ^2^_1_ = 0.96, β = 0.49 (0.51), P = 0.33), or the mating duration of his competitor (Χ^2^_1_ = 1.64, β = −0.69 (0.54), P = 0.20). A non-significant interaction between the mating duration of the focal and competitor male was removed from the final model (Χ^2^_1_ = 1.09, P = 0.29).

The mating duration of the focal male (raised by exponent 0.04) was not affected by the population sex ratio from which a male was derived (Χ^2^_1_ = 2.18, P = 0.14; Table 1), the weight of the focal male (Χ^2^_1_ = 0.83, β = 0.04 (0.04), P = 0.36), or the weight of the female (Χ^2^_1_ = 1.31, β = 0.05 (0.05), P = 0.25).

## Discussion

After 32 generations of experimental evolution, we found no divergence in male investment in testes, accessory gland size or ejaculate weight among populations of *C. maculatus* evolving under a male-biased or female-biased sex ratio. Accordingly, males from male-biased populations did not induce longer female refractory periods, and were not superior sperm competitors. Increased postcopulatory sexual selection via sex-ratio manipulation has been found to generate divergence in male reproductive investment in a range of other species^19^,^20^,^21^,^23^,^24^, and indeed, has generated divergence in female mating behaviour in these same evolution lines^26^. Why increased postcopulatory sexual selection did not generate increased investment into male reproductive traits in this study is unclear.

There are at least four mutually non-exclusive reasons why our experimental manipulations did not generate divergence in male reproductive investment, despite strong theoretical predictions and analogous evidence from other species. First, there may not have been sufficient selection (intensity or duration) to generate divergence in the traits measured. This, however, seems unlikely as a previous study of these same lines demonstrated significant divergence in both physiological and behavioural traits^26^. Males from male-biased lines had reduced immune activity, which, importantly, is an established trade-off with reproductive investment in a range of taxa^34^,^35^,^36^. Sex ratio manipulation also generated divergence in female reproductive behaviour: females from female-biased, but not male-biased lines, were able to respond plastically to socio-sexual cues informing conflict intensity; females from female-biased populations kicked sooner during mating in a male-biased, compared with a female-biased, environment^26^. In their study of *C. maculatus*, Cayetano *et al.*^25^ found divergence in male genital morphology after just 18–21 generations of enforced monogamy. Thus, the absence of divergence in male sperm competitive traits seems unlikely to be due to an insufficient timeframe for selection.

Second, we examined variation in male investment at his first mating, and the effects of selection may be manifest later in an individual’s lifespan. For example, in a similar study, male *D. melanogaster* were reared under male- or female-biased sex ratios over many generations. Experimental males were then mated to five females, in succession. Linklater *et al.*^37^ found that divergence in male reproductive investment between the lines was not evident in male morphology (testes size or accessory gland size), nor was it evident in the male’s first mating attempt: males from male-biased lines did not show the predicted greater reproductive output with the first females. Rather, the increased reproductive investment expected in the male-biased lines was evident only in the rate of declining fertility with subsequent matings: males from male-biased lines showed a faster rate of decline in reproductive investment over consecutive matings. The authors proposed that this was likely due to an increased investment in seminal fluid, as the accessory glands showed a faster rate of depletion in males from male-biased lines^37^. Similarly, also in *D. melanogaster*, Wigby and Chapman^22^ showed that males with an evolutionary history of male-biased sex ratios did not have larger testes or accessory glands than those with an evolutionary history of female-biased sex ratios. Rather, the increased sexual investment manifested itself as an increased mating rate. Furthermore, we only looked at the gross measures of sperm production–testes size and ejaculate weight (a combination of sperm and seminal fluid). Yet, testes size is not necessarily tightly correlated with sperm production^38^. For example, in house mice (*Mus domesticus*), male testes size per se did not respond to postcopulatory sexual selection, but there was a change in the testes architecture that facilitates increased sperm production^39^. Whether there has been divergence in the rate of ejaculate depletion, mating capacity or testes architecture of males among our experimental lines should be the focus of future research.

Third, strategic investment into testes size, sperm number and accessory glands are likely to depend on species-specific patterns of sperm precedence. Under a fair raffle model of sperm utilization we would expect a male’s fertilisation success to be proportional to the number of his sperm represented in the female’s sperm stores^11^. Under such a scenario, increases in ejaculate investment, particularly sperm number, are likely to be favoured. Under a loaded raffle, however, for a range of potential intrinsic and extrinsic reasons, sperm utilization patterns are biased towards a particular ejaculate (for a review, see^11^). Here, selection on increased male investment is likely to be weaker, as the importance of relative mating position supersedes the importance of sperm numbers. Typically, there is strong last-male sperm precedence in *C. maculatus*, which is consistent over a number of female mating frequencies (P_2_ = 0.78; P_3_ = 0.83^40^), and is consistent when male morphological markers, radiation and genetic markers are used to assign paternity^40^,^41^,^42^. High last-male sperm precedence has also been reported in the source population for this experiment^31^. In laboratory populations, males benefit from larger ejaculates: the number of sperm transferred by a male increases his paternity share, but only when mating in the last position^43^. In our experimental populations, however, it is possible that the advantage of larger ejaculates may be diminished by the elevated risk of sperm displacement. Indeed, the last male sperm precedence in this species may explain why a previous study in which postcopulatory sexual selection was manipulated by enforcing either monandry (with no potential for sperm displacement) or polyandry found divergence in male sperm competitive traits^27^, yet our study in which both experimental treatments had the potential for sperm displacement did not.

Interestingly, while our study found significant last-male sperm precedence, it was lower than previous reports^40^,^41^,^42^. Although we did not have an equal sex ratio (control) population in this experiment, it is possible to compare ejaculate traits from our experimental individuals with those from the original source population. Males from our experimental lines had relatively smaller ejaculates for their body weight than source population males (focal male ejaculate weight (mg) = 0.16 ± 0.005; source population males = 0.19 ± 0.01; F_1,164_ = 10.53, P = 0.001)^31^. This comparison must be made cautiously, however, as the source population was maintained on a different diet (black-eyed beans). As relative paternity success can be influenced by the number of sperm contributed by males mating in the last position^43^ (as were the focal males in our experimental design), the potentially reduced ejaculate weight of our experimental males could explain the slightly lower P_2_ values in a species with typically high last male sperm precedence. Nevertheless, given the moderate last-male sperm precedence in our experimental populations, we might predict that postcopulatory selection should act more strongly on the ability of males to prevent females from remating rather than increasing ejaculate/testes size per se. The fact that we also found no evidence of divergence in the female refractory period is perhaps more surprising. Further investigation is clearly required to understand sperm precedence patterns in these experimental populations.

Finally, the strength of postcopulatory sexual selection is typically manipulated by altering the sex ratio or by enforcing monogamy/polygamy. A key disadvantage of manipulating sex ratio is that male reproductive investment in single matings can be entangled with total male reproductive investment^44^. For example, in female-biased populations, sexual selection is reduced and thus, a reduction in male reproductive investment is expected to evolve. However, in female-biased lines, by their very design, there should be an excess of females, and thus increased male mating opportunities. Thus, the increased investment into reproduction that is expected under high sperm competition risk in the male-biased populations may be balanced by the increased investment into testes and accessory glands required to inseminate more females in the female-biased populations–the male mating rate hypothesis (for a review, see^45^). Empirically, this confounding expectation of male investment was explored explicitly in *D. melanogaster*. Reuter *et al.*^46^ created experimental populations that evolved under varying degrees of female sex-bias. They demonstrated that males in highly female-biased lines (in which male mating rate was very high, but sperm competition was low) developed larger testes than moderately female-biased populations (in which male mating rate was relatively lower, but sperm competition relatively higher). In doing so, they demonstrated that increased male reproductive investment was driven by selection for increased male mating rate, and not increased sperm competitiveness. Although a precise measure of male and female mating rates within our experimental evolution lines would be largely impossible to acquire, an indirect estimate of male and female mating capacity would be useful in assessing the merits of each of our explanations for the absence of divergence in our populations.

Accurate predictions regarding the evolution of male investment strategies require an understanding of the precise selective pressures involved. Experimental evolution studies that manipulate adult sex-ratio bias can simultaneously alter the intensity of sexual selection and male mating rates, potentially confounding predictions of how males should invest in reproduction. We suggest caution in using sex ratio manipulation to adjust postcopulatory sexual selection, especially in species where sperm precedence patterns are high or unknown.

## Additional Information

**How to cite this article**: McNamara, K. B. *et al.* Male-biased sex ratio does not promote increased sperm competitiveness in the seed beetle, *Callosobruchus maculatus. Sci. Rep.* **6**, 28153; doi: 10.1038/srep28153 (2016).

## Figures and Tables

**Table 1:** The effect of evolution under different sex ratios on the ability of males to induce a refractory period, male reproductive morphology, mating behaviour, female reproductive output and paternity.

	Male background	
	Male-biased	Female-biased
Duration of refractory period (d)	2.60 ± 0.13 (65)	2.70 ± 0.09 (88)
Ejaculate weight (mg)	0.15 ± 0.006 (62)	0.16 ± 0.008 (83)
Testes size (mm^2^)	0.58 ± 0.01 (99)	0.57 ± 0.01 (103)
Accessory gland size (mm^2^)	0.82 ± 0.02 (50)	0.86 ± 0.02 (51)
Mating duration (s)	466.02 ± 20.17 (63)	534.62 ± 25.52 (64)
Eggs laid	37.17 ± 2.30 (63)	34.26 ± 2.34 (64)
Paternity of second male (P_2_)	0.77 ± 0.04 (60)	0.67 ± 0.04 (62)

Data are means ± standard errors and sample sizes are in parentheses.
